# Autoimmunity-Associated LYP-W620 Does Not Impair Thymic Negative Selection of Autoreactive T Cells

**DOI:** 10.1371/journal.pone.0086677

**Published:** 2014-02-03

**Authors:** Dennis J. Wu, Wenbo Zhou, Sarah Enouz, Valeria Orrú, Stephanie M. Stanford, Christian J. Maine, Novella Rapini, Kristy Sawatzke, Isaac Engel, Edoardo Fiorillo, Linda A. Sherman, Mitch Kronenberg, Dietmar Zehn, Erik Peterson, Nunzio Bottini

**Affiliations:** 1 Division of Cellular Biology, La Jolla Institute for Allergy and Immunology, La Jolla, California, United States of America; 2 Center for Immunology, Department of Medicine, University of Minnesota, Minneapolis, Minnesota, United States of America; 3 Swiss Vaccine Research Institute, Epalinges, and Division of Immunology and Allergy, Department of Medicine, Lausanne University Hospital, Lausanne, Switzerland; 4 Institute for Genetic Medicine, University of Southern California, Los Angeles, California, United States of America; 5 Istituto di Ricerca Genetica e Biomedica (IRGB), CNR, Monserrato, Italy; 6 Department of Immunology, The Scripps Research Institute, La Jolla, California, United States of America; 7 Division of Developmental Immunology, La Jolla Institute for Allergy and Immunology, La Jolla, California, United States of America; Penn State University, United States of America

## Abstract

A C1858T (R620W) variation in the *PTPN22* gene encoding the tyrosine phosphatase LYP is a major risk factor for human autoimmunity. LYP is a known negative regulator of signaling through the T cell receptor (TCR), and murine *Ptpn22* plays a role in thymic selection. However, the mechanism of action of the R620W variant in autoimmunity remains unclear. One model holds that LYP-W620 is a gain-of-function phosphatase that causes alterations in thymic negative selection and/or thymic output of regulatory T cells (T_reg_) through inhibition of thymic TCR signaling. To test this model, we generated mice in which the human LYP-W620 variant or its phosphatase-inactive mutant are expressed in developing thymocytes under control of the proximal *Lck* promoter. We found that LYP-W620 expression results in diminished thymocyte TCR signaling, thus modeling a “gain-of-function” of LYP at the signaling level. However, LYP-W620 transgenic mice display no alterations of thymic negative selection and no anomalies in thymic output of CD4^+^Foxp3^+^ T_reg_ were detected in these mice. *Lck* promoter-directed expression of the human transgene also causes no alteration in thymic repertoire or increase in disease severity in a model of rheumatoid arthritis, which depends on skewed thymic selection of CD4^+^ T cells. Our data suggest that a gain-of-function of LYP is unlikely to increase risk of autoimmunity through alterations of thymic selection and that LYP likely acts in the periphery perhaps selectively in regulatory T cells or in another cell type to increase risk of autoimmunity.

## Introduction

The *PTPN22* gene, encoding the lymphoid tyrosine phosphatase LYP, has emerged as one of the major non-HLA risk factors for a wide range of autoimmune diseases, including type 1 diabetes, rheumatoid arthritis (RA), systemic lupus erythematosus, Graves’ disease and others [1], [2]. A missense *C1858T* single nucleotide polymorphism in exon 14 of the *PTPN22* gene leads to LYP-R620W substitution. The variant *PTPN22* allele confers to carriers a roughly two-fold increased risk of autoimmunity [2]–[5]. LYP inhibits signaling through the T cell receptor (TCR), and its substrates in T cells include the phosphorylated tyrosine residues in the activation motifs of Lck, Zap-70 and other signaling molecules [4], [6]–[8]. Mice made deficient for *Ptpn22* (encoding Pep, the murine LYP-homolog PEST-enriched phosphatase) display a phenotype of increased TCR signaling in effector T cells, which correlates with an expansion of the effector-memory T cell compartment [9], [10]. The LYP-R620W substitution impairs the ability of the phosphatase to bind to the SH3 domain of the C-terminal Src-family kinase CSK [3], [4], which is a major LYP interactor in T cells [7], [11]. LYP-W620 also displays 1.5–2 fold increased intrinsic phosphatase activity compared to the common R620 variant [12]–[14].

Studies of the effect of the LYP-R620W substitution on immune cell signaling have not yet yielded a unifying model. We and others reported that TCR signaling is impaired in T cells from patients with autoimmune disease who carry the LYP-W620 variant [12], [15]–[17]. Reduced signaling through antigen receptors has also been reported in B cells and peripheral blood mononuclear cells (PBMC) of both patient and healthy donor LYP-W620 carriers [13], [15], [18]. Together, these findings suggest that the LYP-W620 variant is a “gain-of-function” negative regulator of antigen receptor signaling.

Several models have been proposed to explain the gain-of-function phenotype, including increased phosphatase activity following reduced CSK-mediated phosphorylation of the regulatory Tyr536 residue [14], and increased recruitment of the LYP-W620 variant to lipid rafts following release from cytoplasmic sequestration by Csk [19]. However, others have proposed an opposing model wherein the R620W substitution confers “loss-of-function” effects on antigen receptor signaling. Supporting data for a LYP-W620 “loss-of-function” hypothesis come from overexpression experiments in Jurkat T cells [20]. Enhanced TCR-driven calcium mobilization was observed in human LYP-W620 carriers and in T cells from a mouse carrying a knock-in R619W mutation in mouse Pep that is homologous to the human LYP R620W variation [21]. Chang *et al.* identified a new dominant-negative isoform of LYP and proposed a model that reconciles “gain-of-function” and “loss-of-function” observations [22]. Dai *et al.* recently reported a phenotype of enhanced TCR signaling and spontaneous autoimmunity in R619W knock-in mice [23]. Analysis of the spectrum of phosphorylated molecules in TCR-stimulated Pep-R619W T cells suggested altered enzymatic specificity [23]. In line with this “altered function” model, a recent analysis of peripheral T cells from genotyped healthy subjects suggested that the LYP-R620W mutation can positively or negatively affect TCR signaling, depending on the biochemical readout assayed and on the stage of signaling [24].

A prevailing model of thymocyte selection holds that TCR affinity for MHC/peptide ligand plays a central role in shaping the peripheral TCR repertoire [25]. Deletion of autoreactive thymoyctes and agonist selection of regulatory T cells (T_reg_) are two important mechanisms for establishing T cell tolerance that depend upon high-affinity interactions between TCR and ligand.

Phenotyping of *Ptpn22* knockout mice suggested that Pep might play a role in regulating thymic selection. Increased positive thymic selection has been reported in *Ptpn22* KO mice [9] and in two independently-generated Pep R619W knock-in mouse models, one of which developed spontaneous autoimmunity [21], [23]. Increased negative selection of H-Y transgenic male thymocytes mice was reported in one Pep R619W knock-in model [23], but thymic deletion was not altered in the other Pep R619W knock-in strain [21] or in *Ptpn22* KO mice [9]. We reported increased TCR signaling in *Ptpn22* KO thymocytes, correlating with increased thymic output of T_reg_ in KO mice [26]; however, another group found no difference in thymic T_reg_ percentages in an independently generated KO model [23]. The potential effect of the autoimmune-associated human LYP-W620 variant in thymic selection has not yet been examined in a controlled genetic environment (congenic mice). Yeh *et al*. recently reported that overexpression of wild type Pep under control of the distal *Lck* promoter did not result in alteration of thymocyte numbers in the NOD background [27], however the distal *Lck* promoter is expressed only in late stages of thymocyte selection [28], and the effect of the autoimmune-predisposing R620W variant was not assessed.

In this study, we addressed effects of the human LYP-W620 variant on thymocyte signaling and selection by expressing the variant phosphatase under control of the proximal *Lck* promoter. To model effects of altered enzymatic activity inherent in LYP-W620, we studied mice exhibiting low overexpression levels of the active human phosphatase in thymocytes. To detect possible effects unrelated to the increased phosphatase activity, we also examined animals overexpressing an enzymatically-inactive mutant (C227S) of the LYP-W620 phosphatase. Mice transgenic for the active phosphatase displayed reduced thymocyte TCR signaling when compared to mice transgenic for the inactive phosphatase or to non transgenic littermates. However no significant alterations of T cell selection or of thymic T_reg_ output were observed in LYP-W620 transgenic mice. Our study suggests that the reported gain-of-function activity of LYP-W620 is insufficient to significantly alter thymic output; thus it is unlikely that its major disease-predisposing role is exerted during thymocyte selection.

## Materials and Methods

### Mouse Experimental Work

#### Generation of Prox*Lck*-driven transgenic mice

cDNAs encoding either N-terminal HA-tagged LYP-W620 (LYPW) or its inactive C227S mutant (LYPW^C227S^) were cloned into the *BamHI* site of the *p*1017 vector [29](obtained from T. Mustelin, Sanford-Burnham Medical Research Institute). The transgenes were isolated from the vector backbone by digestion with *Not*I, and pronuclear oocyte injection was performed using fertilized ova from C57BL/6 (B6)D2F1 females, and pseudopregnant outbred ICR females as foster mothers. Genotyping of transgenic founders and all transgenic animals was performed by PCR, using one primer 5′-tgtgaacttggtgcttgagg-3′ on the *Lck* promoter and another 5′-tgttatggcatgcatggagt-3′ on the LYP cDNA. All founder animals were bred with C57BL/6Tac mice and germline transmission was obtained from three B6D2F1-Tg(LckproxLYP-W620)/Igm founders and two B6D2F1-Tg(LckproxLYP-W620/S227)/Igm founders. Lines were generated from each of the founders carrying the transgenes in hemizygosity. Each line was tested for expression of the transgene and activity of the transgenic phosphatase. The two founder lines B6D2F1-Tg(LckproxLYP-W620)958/Igm (here abbreviated as TgLYPW) and B6D2F1-Tg(LckproxLYP-W620/S227)963/Igm (here abbreviated as TgLYPW^C227S^) were selected for further studies and extensively backcrossed onto the BALB/c background for >15 generations before breeding with Skg mice (see below).

#### Generation of BAC-transgenic mice

Recombineering (homologous recombination in bacteria) [30] was used to introduce the autoimmune-associated variant (*PTPN22-C1858T*) into the human *PTPN22* locus. A 200 kb Bacterial Artificial Chromosome (BAC; Invitrogen) fragment of human genomic DNA containing the entire *PTPN22* locus, including 60 kb located 5′ to the *PTPN22* transcriptional start site, was used as a template. Following recombineering, 116 kb *NotI* restriction fragments encoding the disease variant LYP-W620 protein was used for pronuclear B6 oocyte injection and generation of founder mice (BACLYPW) directly on B6 background as described above. LYP expression in BACLYPW mice has been described elsewhere [31].

#### Skg mice and arthritis scoring

Skg mice were kindly provided by S. Sakaguchi (Kyoto University, Kyoto, Japan). Skg/Skg mice are homozygous for a loss-of-function W163C mutation of Zap-70 that leads to decreased thymic TCR signaling and altered selection of self-reactive CD4^+^ T cells and of T_reg_ [35], [36]. These mice display alterations of the Vβ thymic repertoire and develop spontaneous arthritis on the BALB/c background [35], [37], [38], whose frequency and clinical course is dependent on microbial colonization and on cleanliness of the environment. However severe arthritis can be induced in all Skg/Skg mice, independent on their microbial colonization, by intraperitoneal injection with fungal wall polysaccharides, including zymosan and mannan [39], [40]. In the La Jolla Institute for Allergy & Immunology (LJI) animal facility, Skg/Skg animals developed spontaneous arthritis with very low frequency and the severity of arthritis was variable among cages. However all Skg/Skg mice developed severe arthritis after intraperitoneal injection of 20 mg mannan (purchased from Sigma) dissolved in 200 µl PBS. Skg/WT heterozygous mice display altered thymic selection [36], [37] and alterations of the Vβ thymic repertoire [37] qualitatively similar to the ones found in Skg/Skg mice, however they do not develop spontaneous [35] or polysaccharide-induced (our unpublished observation) arthritis. Clinical scoring of arthritis was carried out as described [35].

#### Other mouse models

Foxp3^GFP^ mice were kindly provided by A. Rudensky (Sloan-Kettering Institute, NY) [32]. HY-TCR mice [33] were kindly provided by H. Cheroutre (La Jolla Institute for Allergy and Immunology, CA), or by K. Hogquist (University of Minnesota; for the crosses with BACLYPW mice), and Vβ5 TCRβonly transgenic mice by P. Fink (University of Washington, WA). BALB/c and DO11.10 mice [34] were purchased from Taconic and Rip-mOva and OT-1 mice from Jackson laboratories. *Ptpn22*-deficient mice were kindly provided by A. Chan (Genentech).

#### Bone marrow chimeras and infections with Lm-Ova and VSV-Ova

Recipient mice were lethally irradiated with 900 rad and one day later engrafted with bone marrow cells isolated from the femur and tibia. Prior to injection, the bone marrow was T cell depleted by incubating cells with biotinylated anti-CD3 antibody (eBiociences), anti-biotin microbeads followed by magnetic separation in LS columns (all Miltenyi Biotec, Bergisch-Gladbach, Germany). Mice were treated with antibiotics (Bactrim, Roche) until 3 weeks post-irradiation.

Frozen stocks of recombinant, ovalbumin expressing *Listeria monocytogenes* [Lm-Ova] were grown in brain-heart infusion broth (Oxoid, UK) to mid log phase. Bacterial numbers were determined by measuring the OD at 600 nm. 1000–3000 cfu were injected in PBS intravenously. *Vesicular stomatitis virus* expressing Ova (VSV-Ova) [41] were grown and titered on BHK cells. Frozen stocks were diluted in PBS to 2×10^6^ pfu and injected intravenously.

### *Ex vivo* Thymocyte or T cell Stimulation

Spleen and thymus cell suspensions were obtained by mashing the organs through a 100 µm nylon cell strainer (BD Falcon). Red blood cells were lysed with a hypotonic ACK lysis buffer. 5×10^5^ CD45.1 congenic thymocytes were co-cultured in 96 well plates for 18–22 hours with 7.5×10^4^ irradiated RMA cells and titrated doses of SIINFEKL, SIITFEKL, or SIIVFEKL peptide (EMC Microcolelctions, Tübingen, Germany). Cultures were stained with anti-CD8 (53–6.7, PerCP, eBioscience), CD45.1 (clone A20), CD4 (GK1.4) purchased from BioXcell (West Lebanon, NH) and conjugated to FITC or A647 using labeling reagents from Invitrogen.

To identify K^b^/Ova reactive T cells in infected mice, up to 4×10^6^ total splenocytes were seeded into 96 well plates. The cells were stimulated with titrated doses of SIINFEKL peptide for 30 min at 37°C, then 7 µM Brefeldin A (Sigma) was added and the cells were returned to 37°C for another 4.5 hours.

Afterwards, the cells were washed, incubated for 30 min at 4°C in PBS supplemented with 2% FBS and 0.01% azide (FACS buffer) and then stained with the following antibodies: anti-CD8 PerCP-Cy5.5 (clone 53–6.7, eBioscience), anti-CD4 and anti-IFNγ (BioXcell, self-conjugated to FITC, Invitrogen). Then the cells were fixed and permeabilized using the Cytofix/Cytoperm Kit (BD, Franklin Lakes NJ, USA) and stained with anti-IFNγ (BioXCell, self-conjugated to Alexa Fluor 647, Invitrogen). Data were analyzed with FlowJo software (Tree Star Inc). Graphs were prepared and EC_50_ concentrations were determined with GraphPad Prism.

Stimulation for the CD69 expression assay was performed by precoating 96-well plates with 10 µg/ml and 25 µg/ml anti-CD3 overnight at 4°C and then culturing thymocytes at 2×10^5^ cells/well for 18 h at 37°C.

### Phospho-Erk (pErk) ELISA

pErk levels were detected by ELISA using the PathScan® Phospho-p44 MAPK (Thr202/Tyr204) Sandwich ELISA antibody pair (Cell Signaling Technology) according to manufacturer’s protocols.

### FACS Analyses and Intracellular Staining (ICS)

Abs (conjugated to eFluor450, Pacific Blue, APC, PE, FITC, PE-Cy7, PerCP-Cy5.5, PerCP-eFluor710, V500 and AlexaFluor700) directed against the following surface markers were obtained from eBioscience, BD PharMingen, BioLegend, Proimmune and used at a 1/100 dilution to analyze immune cell subsets in single cell suspensions of thymi and lymph nodes of LYP Tg mice: CD3ε (145-2C11), CD4 (GK1.5), CD8α (53-6.7), CD25 (PC61.5), CD44 (IM7), CD69 (H1.2F3), B220/CD45R (RA3–6B2), Vβ3 (KJ25), Vβ5.1/5.2 (MR 9-4), Vβ8.1, 8.2 (KJ16), DO11.10 TCR (KJ1-26), HY TCR (T3.70), CD1d tetramers. For intracellular cytokine staining [40], lymph node (LN) cells were stimulated with 20 ng/ml PMA and 1 µM ionomycin in the presence of GolgiStop (BD Biosciences) for 5 h. Cells were then stained for surface antigens, fixed, and permeabilized using Cytofix/Cytoperm buffer (BD Biosciences), followed by anti-IL-17 (BD Pharmingen) staining. To detect low level expression of HA-tagged LYP by ICS indirect immunofluorescence was used to increase sensitivity. Thymocytes were first stained for surface markers followed by fixation and permeablization. Cells were then stained with an anti-HA Ab (Cell Signaling Technology) for 1 hour followed by washing and staining with FITC-conjugated anti-rabbit Ab (BD PharMingen). For pErk ICS, cells were stimulated with 10 µg/ml anti-CD3 for 20 min, followed by fixation in 4% paraformaldehyde. After washing, cells were permeabilized with 90% methanol at 4°C, then stained with anti-phospho-p44/42 MAPK (Erk1/2) (Cell Signaling Technology) followed by washing and staining with FITC-conjugated anti-rabbit Ab (BD Pharmingen). All flow cytometry experiments were performed using an LSRII (BD Bioscience) and data was analyzed using FlowJo Software (Tree Star).

### Immunoprecipitations

For immunoprecipitations (IPs), cells were lysed in 20 mM Tris/HCl, pH 7.4, 150 mM NaCl, 5 mM EDTA with 1% Nonidet P-40, 1 mM phenylmethylsulfonyl fluoride, 10 µg/ml aprotinin, 10 µg/ml leupeptin and 10 µg/ml soybean trypsin inhibitor. Lysates were probed with the indicated antibodies for 2 h at 4°C, followed by incubation with PGSepharose beads (GE Healthcare) for 1 h at 4°C. Beads were then washed in lysis buffer, and proteins were eluted in Laemmli sample buffer (Bio-Rad).

### Quantitative PCR (qPCR)

Total human *PTPN22* and mouse *Ptpn22* mRNA expression was measured by quantitative real-time RT-PCR (qPCR) using specific primers designed to amplify the human and mouse mRNAs. Total RNA was isolated using the RNeasy Plus Micro Kit (Qiagen). After DNase treatment of the RNA with Ambion® TURBO™ DNase (Life Technologies), cDNA was reverse transcribed using the SuperScript® III First-Strand Synthesis System (Life Technologies). Gene expression was quantified using SYBR Green reaction mixture (SaBiosciences/Qiagen) on a LightCycler 480 (Roche) with a cycling program of 94°C for 3 minutes, 35 cycles of (94°C for 45 seconds, 62°C for 30 seconds, 72°C for 1 minute), 72°C for 10 minutes. Reactions were performed in triplicate and normalized to the mouse housekeeping gene RNA Polymerase II (Polr2a). Primer sequences were:

PTPN22 forward 5′-GAATTTCTGAAGCTGAAAAGGCA-3′

PTPN22 reverse 5′-TCCCAAATCATCCTCCAGAAGTC-3′

Polr2a forward 5′ CCAGGAAACACATTGCGTC-3′

Polr2a reverse 5′-GGAAGAAGAACTCAGTGGGTG-3′.

### Phosphatase Assays

Phosphatase assays were performed on HA-tagged LYP following anti-HA-IPs from thymocytes from the indicated mice. Cells were lysed in 50 mM HEPES, pH 7.4, 150 mM NaCl, 5 mM EDTA with 1% Triton-X 100, 1 mM phenylmethylsulfonyl fluoride, 10 µg/ml aprotinin, 10 µl/ml leupeptin, and 10 µg/ml soybean trypsin inhibitor. The IPs were washed in 50 mM Bis-Tris, pH 6.0, and then resuspended in phosphatase buffer (50 mM Bis-Tris, pH 6.0, 5 mM DTT, 0.005% Tween-20, 5 mM sodium fluoride, 2 mM sodium pyrophosphate). After the addition of 0.1 mM 6,8-Difluoro-4-Methylumbelliferyl Phosphate (DiFMUP, Life Technologies), the reaction was monitored continuously by measuring the increase in fluorescence (λ_ex_ = 340 nm and λ_em_ = 460 nm) at 60 s intervals for 30 min using a Tecan Infinite M1000 plate reader (Tecan) [42]. The activity measured in triplicate was corrected for the nonspecific signal of identical reactions performed also in triplicate without the addition of enzyme.

#### Ethics statement

This study was carried out in strict accordance with the recommendations in the Guide for the Care and Use of Laboratory Animals of the National Institutes of Health. The protocol was approved by the institutional Animal Use Committees of the University of Southern California (Protocols #19853 and #10714), the La Jolla Institute for Allergy and Immunology (Protocol #AP140-NB4), the University of Minnesota (Protocol #1106A00402) or the veterinarian authorities of the Swiss Canton Vaud (Protocols 2253 and 2643). All efforts were made to minimize animal suffering.

## Results

### Generation of Mice Carrying Thymic Overexpression of LYP-W620

**Figure 1:**
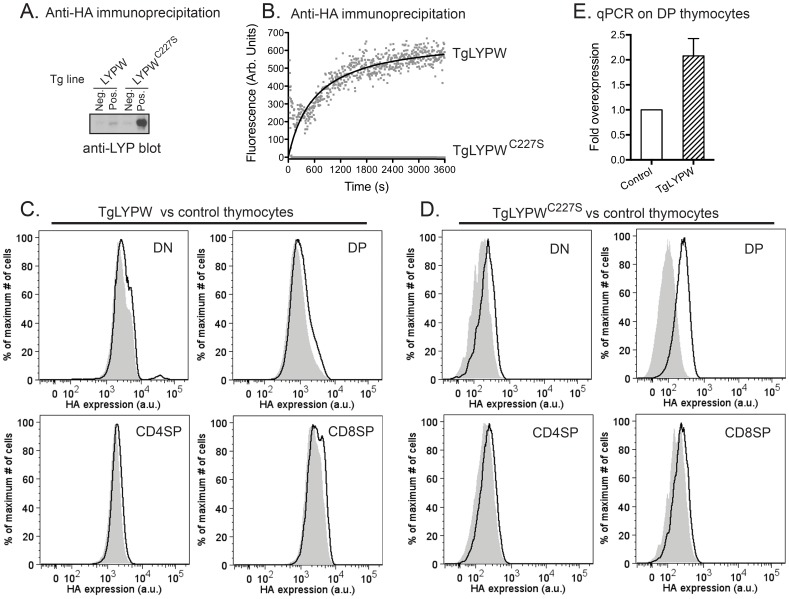
Mice transgenic for LYP-W620 (TgLYPW) show overexpression of LYP in DP thymocytes. A, LYP protein expression in TgLYPW thymocytes. Total thymocytes from TgLYPW (lane 2) or a non-Tg littermate (lane 1) and TgLYPW^C227S^ (lane 4) or a non-Tg littermate (lane 3) were lysed and subjected to immunoprecipitation (IP) using an anti-HA antibody (Ab). Panel shows Western blotting using an anti-LYP Ab. Data are representative of 3 independent experiments with similar results. B, LYP phosphatase activity in TgLYPW thymocytes. Anti-HA IPs were performed from lysates of total thymocytes from TgLYPW and TgLYPW^C227S^ mice. Graphs show the phosphatase activity of LYP as assessed by dephosphorylation of the fluorescent substrate DiFMUP. Data are representative of 2 independent experiments with similar results. C–D, LYP-W620 transgene expression in thymocyte subpopulations. Expression of LYPW (C) or LYPW^C227S^ (D) was assessed by intracellular staining using a fluorophore-conjugated anti-HA Ab in DN (upper left panel), DP (upper right panel) CD4SP (lower left panel) and CD8SP (lower right panel) thymocytes from Tg mice (black graphs) and control non-Tg littermates (grey filled graphs). E, Quantification of overexpression of LYPW relative to endogenous Pep in DP thymocytes of TgLYPW mice. mRNA encoding LYP and Pep was quantified by qPCR from sorted DP thymocytes from control BALB/c (white bar) and TgLYPW (striped bar) mice, using a primer pair that amplifies both human *PTPN22* and mouse *Ptpn22* mRNAs. Graph shows relative expression levels of total *PTPN22* after normalization to the mouse housekeeping gene *Polr2a*. Data are average and SE of 3 biological replicates.

In order to assess the effect of LYP-W620 overexpression at the thymic level, we generated transgenic mouse lines overexpressing N-terminal HA-tagged LYPW (abbreviated as TgLYPW) or its inactive mutant LYPW^C227S^ (abbreviated as TgLYPW^C227S^) under the control of the proximal *Lck* promoter. The *Lck* proximal promoter is highly active in thymocytes, including immature double negatives (DN), double positives (DP), and CD4^+^ and CD8^+^ single positives (SP) [29], but is repressed after thymocyte emigration. **Fig. 1A** shows that the two transgenic constructs were expressed in thymocytes, although at different levels. Progeny of an additional independent founder expressing TgLYPW^C227S^ all displayed overexpression levels several orders of magnitude higher than those observed in the TgLYPW line expressing the active phosphatase (data not shown). A single TgLYPW^C227S^ founder line was selected for all further analyses. In line with known thymus-specific activity of the proximal *Lck* promoter, no expression of the transgenes was detectable in the spleen and lymph nodes of the transgenic mice (data not shown). The transgene-encoded LYP-W620 protein was active in an *in vitro* phosphatase assay, while as expected, LYP-W620/S227 was completely inactive (**Fig. 1B**). The highest expression of the transgenic protein was achieved in DP thymocytes, although overexpression was evident in SP and DN cells (**Fig. 1C** and **Fig. 1D**). An analysis at the mRNA level using primers that cross-react with endogenous *Ptpn22* suggested that thymocytes from the TgLYPW line carry an average two-fold overexpression of *PTPN22* transcript (**Fig. 1E**).

### Thymic Overexpression of LYPW Leads to Reduced Thymocyte TCR Signaling

**Figure 2:**
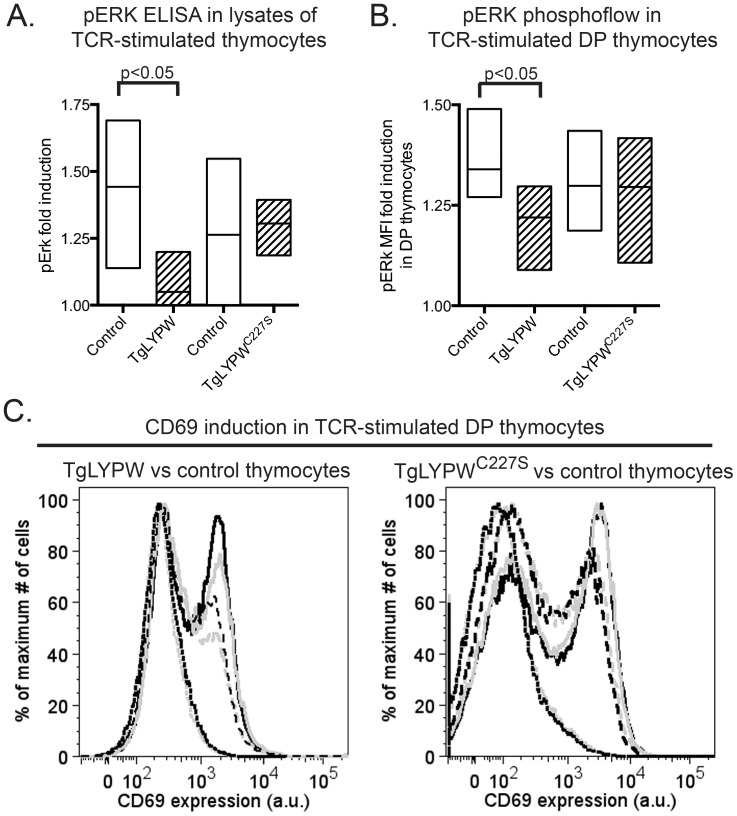
Overexpression of LYPW inhibits TCR signaling in DP thymocytes. A–B, Overexpression of LYPW causes reduced activation of Erk in thymocytes. Total thymocytes from TgLYPW or TgLYPW^C227S^ (striped bars) or their respective non-Tg littermates (white bars) were stimulated with 20 µg/ml anti-CD3 and 10 µg/ml anti-Armenian Hamster IgG1 crosslinker for 2.5 minutes. A, Graph shows phosphorylation of Erk in total thymocyte lysates assessed using the PathScan® phospho-p44 MAPK (Thr202/Tyr204) sandwich ELISA kit. Histogram shows mean and range of fold induction of at least 3 biological replicates. B, Phosphorylation of Erk in DP thymocytes was assessed by phosphoflow analysis after intracellular staining with an anti-pErk Ab. Fold induction of Erk phosphorylation was normalized within each experiment relative to the sample with the highest induction. Histogram shows mean and range of at least 3 biological replicates. C, Overexpression of LYPW causes reduced T cell activation in DP thymocytes. Thymocytes from TgLYPW or TgLYPW^C227S^ (grey graphs) and their respective non-Tg littermates (black graphs) were cultured in the presence of 10 µg/ml (long dashed graphs) or 25 µg/ml (solid graphs) anti-CD3, or media alone (dotted graphs), for 18 hours. Graphs shows expression of CD69 in DP thymocytes as assessed by flow cytometry analysis after staining with an anti-CD69 Ab. Median fluorescence intensity (MFI) values are indicated on each graph. Graphs are representative of at least 3 biological replicates with identical results.

The gain-of-function model predicts that LYP-W620 will exert augmented negative regulatory function in antigen receptor signaling. We therefore assessed whether overexpression of LYPW results in decreased thymocyte TCR signaling. **Fig. 2A** and **Fig. 2B** show that Erk phosphorylation after engagement of the TCR was reduced in thymocytes from TgLYPW transgenic animals compared to cells from non transgenic littermates. Notably, despite exhibiting significantly higher expression of LYP protein compared to TgLYPW mice (**Fig. 1A**), TgLYPW^C227S^ transgenic thymocytes displayed no alteration in Erk phosphorylation compared to non transgenic cells. Similarly, TCR-dependent CD69 upregulation was reduced on DP thymocytes from TgLYPW, but not from TgLYPW^C227S^, transgenic mice, compared to their respective littermates (**Fig. 2C** **)**. Overall, these data indicate that thymocyte-expressed LYP-W620 can repress TCR signaling in total thymocytes and in DP thymocytes in particular, and suggest that modulation of TCR signaling by LYP-W620 is phosphatase-activity dependent.

### Thymic Overexpression of LYPW does not Affect Positive or Negative Selection or the Output of Thymic CD4^+^Foxp3^+^ T_reg_

**Figure 3:**
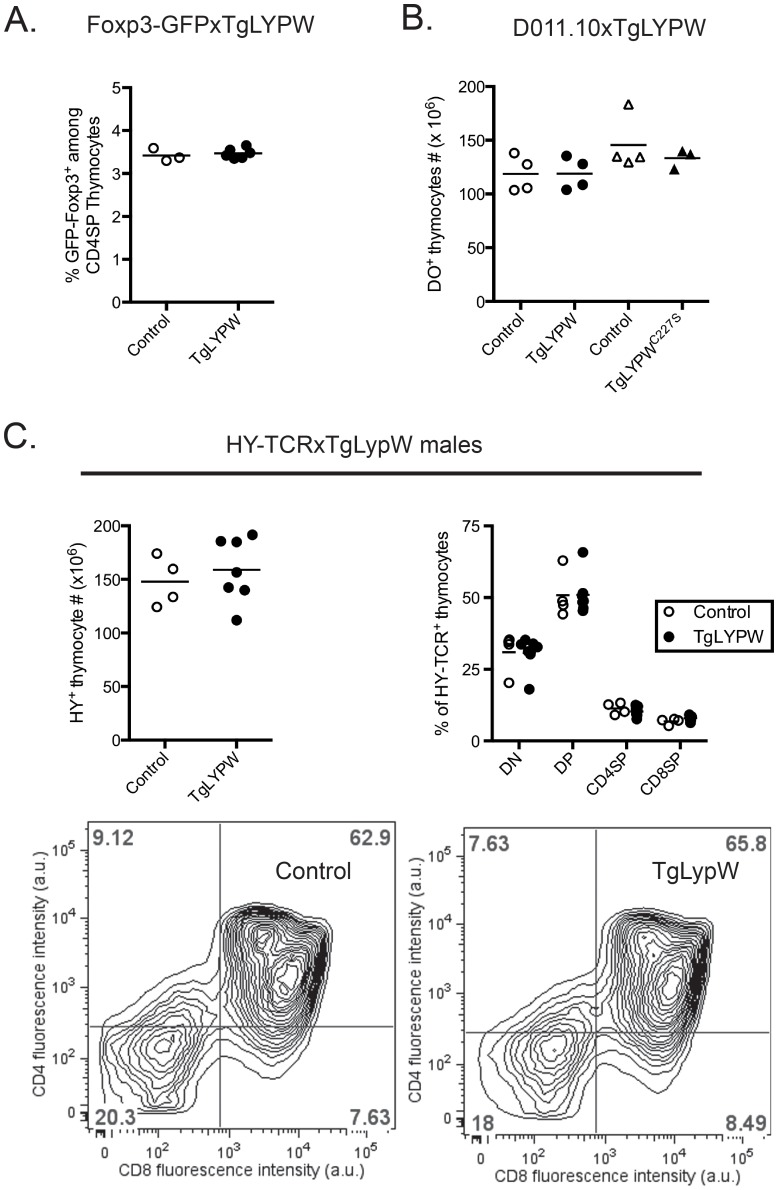
No decrease in thymic output of T_reg_ and positive or negative selection of HY-TCR-transgenic thymocytes in human LYP-W620 transgenic (TgLYPW) and control mice. A, LYP-W620 overexpression does not alter the numbers of CD4^+^Foxp3^+^ thymocytes. Panel shows % of GFP^+^ thymocytes among CD4SP from Foxp3-GFPxTgLYPW mice (black circles, n = 6) or non-Tg littermates (white circles, n = 3). B, No alterations of positive selection of DO11.10^+^ thymocytes in TgLYPW mice. Panel shows numbers of DO11.10^+^ thymocytes in DO11.10xTgLYP^WT^ mice or DO11.10xTgLYP^C227S^ mice (black circles and triangles, n = 4 and n = 3 respectively) or their respective non-Tg littermates (white circles and triangles, n = 4 and n = 4 respectively). C, Overexpression of LYP does not alter negative selection in male F1 HY-TCRx.TgLYP mice. Upper left panel shows numbers of HY-TCR^+^ thymocytes from male F1 HY.TgLYP mice (black circles, n = 7) or non-Tg littermates (white circles, n = 4). Upper right panel shows % of CD4SP, DP, CD8SP and DN thymocytes after gating on the HY^+^ population of thymocytes from male HY-TCRxTgLYPW mice (black circles, n = 4) or non-Tg littermates (white circles, n = 4). Bottom panels shows representative contour plot of CD4^+^ vs CD8^+^ expression of HY-TCR^+^ thymocytes from HY-TCRxTgLYPW or non-Tg littermates. Transversal bars in each panel indicate mean value.

Loss of Pep results in increased numbers of both CD4SP and CD8SP thymocytes, but no defects in negative selection [9], [21]. We asked whether thymocyte expression of LYP-W620 could modulate positive or negative selection. We found no significant alterations in the total thymocyte numbers and numbers of DN, DP, CD4SP, CD8SP, NKT or gamma-delta T cells associated with TgLYPW or TgLYPW^C227S^ expression (**Table 1**). Further, we observed no differences in the percentages of CD4^+^Foxp3^+^ T_reg_ present in the thymi of TgLYPW or TgLYPW^C227S^ mice (data not shown). To further determine whether CD4^+^Foxp3^+^ T_reg_ thymic output is affected by the expression of the LYPW transgene, we bred the transgenic mice with GFP-Foxp3 mice carrying a GFP cassette knocked into the *Foxp3* locus [32]. **Fig. 3A** shows that the frequency of GFP-Foxp3^+^ thymocytes was unaffected by the expression of the LYPW transgene. To address the roles of LYP-W620 and of LYP enzymatic activity in TCR-driven thymocyte development, we bred the transgenic mice to DO11.10 and H-Y TCR transgenic strains that served as models of positive and positive/negative selection, respectively. **Fig. 3B** shows that the numbers of DO11.10 TCR transgenic cells were not affected by expression of the LYPW or LYPW^C227S^ transgenes pointing to the absence of anomalies of positive selection in these mice. As shown in **Fig. 3C**, total numbers of transgenic HY-TCR thymocytes were unaffected by the expression of the active phosphatase in F1 B6xBALB/c male mice. In this model, deletion of the HY-TCR transgene was minimal, likely due to the B6xBALB/c genetic background, however no skewing of the percentages of DP, CD8SP or CD4SP could be found, suggesting that negative selection of CD8^+^ cells operates normally in these mice (**Fig. 3C**). Similarly, we did not see any differences between non transgenic and TgLYPW OT-1 TCR transgenic mice in the efficiency of CD8^+^ negative selection upon crossing the animals to cognate antigen-expressing Rip-mOva mice (data not shown).

**Table 1:** Cell distribution in thymus of TgLYPW and TgLYPW^C227S^.

	TgLYPW	
	TgLYPW (n = 5)	Non Tg littermates (n = 6)
Total thymocyte (x10^6^)	127±46.77	154±32.21
DN	3.12±1.97	2.49±1.07
DP	75.18±1.95	75.13±5.25
CD4SP	13.2±1.84	13.45±2.24
CD8SP	3.45±0.8	3.7±0.69
CD25^−^CD44^+^ % of DN	15.04±3.59	18.22±5.40
CD25^+^CD44^+^ % of DN	4.68±2.05	4.56±2.19
CD25^+^CD44^−^ % of DN	42.16±4.41	39.9±4.03
CD25^−^CD44^−^ % of DN	38.1±7.49	37.35±3.63
NKT cells	1.3±0.19	1.36±0.06
gammadelta T cells	0.27±0.04	0.31±0.03
	**TgLYPW^C227S^**	
	**TgLYPW^C227S^ (n = 5)**	**Non Tg littermates (n = 5)**
Total thymocyte (x10^6^)	160.78±23.99	131.23±21.36
DN	4.32±2.51	3.53±1.26
DP	71.78±6.41	74.78±2.37
CD4SP	17.74±5.02	15.18±2.51
CD8SP	4.13±0.75	4.60±0.73
CD25^−^CD44^+^ % of DN	16.32±9.46	17.16±8.71
CD25^+^CD44^+^ % of DN	5.31±1.65	4.50±2.37
CD25^+^CD44^−^ % of DN	41.96±6.21	37.80±3.33
CD25^−^CD44^−^ % of DN	37.02±3.34	40.60±5.95

Table shows the basic statistics (average±SD) of cell counts in transgenic animals and non transgenic littermates.

**Figure 4:**
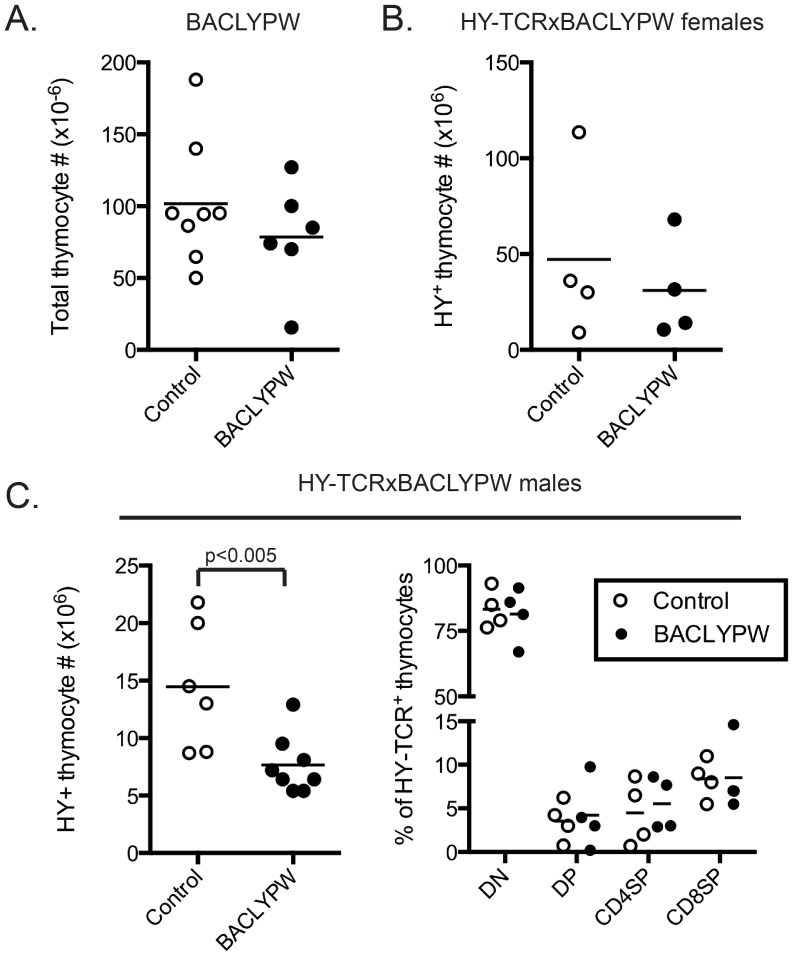
No decrease in positive or negative selection of HY-TCR-transgenic thymocytes in human *PTPN22* regulon transgenic (BACLYPW) and control mice. A, Overexpression of LYP-W620 under control of the *PTPN22* physiological promoter does not affect total thymocyte number in BAC transgenic mice. Panel shows total numbers of thymocytes in BACLYPW mice (black circles, n = 6) or their respective non-Tg littermates (white circles, n = 8). B–C, overexpression of LYP-W620 does not alter positive or negative selection and the subpopulation distribution of HY-TCR^+^ thymocytes in BACLYPW mice. B, panel shows numbers of HY-TCR^+^ thymocytes from female HY-TCRxBACLYPW mice (black circles, n = 4) or non-Tg littermates (white circles, n = 4). C, Left panel shows numbers of HY-TCR^+^ thymocytes from male HY-TCRxBACLYPW mice (black circles, n = 8) or non-Tg littermates (white circles, n = 6). Right panel shows % of CD4SP, DP, CD8SP and DN thymocytes after gating on the HY^+^ population of thymocytes from male HY-TCRxBACLYPW mice (black circles, n = 4) or non-Tg littermates (white circles, n = 4). Transversal bars in each panel indicate mean value.

The above studies of negative selection were carried out in F1 B6xBALB/c mice in which overexpression or mis-expression of *Lck* promoter-directed LYP-W620 might obscure phosphatase-dependent changes in TCR-dependent thymocyte development. Therefore, we sought independent confirmation of our findings by carrying out studies of animals made transgenic for human *PTPN22* on a pure B6 background. Mice bearing an entire *PTPN22*-*T1858 human regulon encoding a LYP-W620 protein were generated on a B6 background under the supposition that endogenous *PTPN22* transcriptional regulatory elements would induce physiologic spatio-temporal expression patterns. To mimic the LYP-W620 phosphatase allele “load” observed in the most common human carrier state (heterozygosity for one allele each of LYP-R620 and LYP-W620), we bred the animals with *Ptpn22*-deficient mice to generate Pep^+/−^.LYP-W620 (abbreviated as BACLYPW) mice. A non-significant trend toward a decrease in the total number of thymocytes was observed in BACLYPW mice compared to non transgenic littermate mice. We observed no differences between Pep^+/−^ littermates and BACLYPW transgenic mice in thymocyte number or distribution (**Fig. 4A** and data not shown). These findings provided further evidence that LYP-W620 expression does not cause grossly altered thymic development.

We also asked whether endogenous promoter-driven LYP-W620 expression affects thymocyte positive or negative selection. We bred BACLYPW mice to H-Y TCR transgenic mice. H-Y TCR transgenic female thymi displayed no significant differences between BACLYPW and non transgenic Pep^+/−^ in total thymocyte numbers or the distribution of H-Y TCR-high cells (**Fig. 4B** and data not shown). A small decrease in the total number of thymocytes was noticed in BACLYPW vs control male HY-TCR transgenic mice (**Fig. 4C**). However, the distribution of the major thymocyte subpopulations remained unaffected in BACLYPWxHY-TCR male mice, compared with age-matched controls (**Fig. 4C**). Together, these data supported the suggestion, first raised by findings in TgLYPW animals (**Fig. 3A**), that LYP-W620 does not differentially regulate thymocyte positive selection. Significantly, the data from H-Y males suggest that if LYP-W620 does modulate negative selection, it does not impair that process. Rather, the presence of human promoter-controlled LYP-W620 may serve to promote deletion of autoreactive thymocytes.

**Figure 5:**
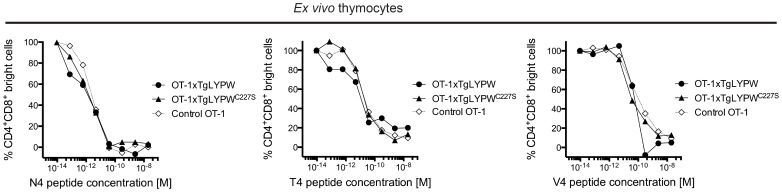
OT-1 thymocytes overexpressing LYP-W620 show similar antigen sensitivity as non-Tg thymocytes. Freshly isolated OT-1 transgenic thymocytes from TgLYP (black circles), TgLYP^C227S^ (black triangles) or control non-Tg mice (white diamonds) were co-cultured overnight together with RMA cells and the indicated doses of SIINFKL peptide (N4, left panel), of the low and very low affinity altered peptide ligands SIITFEKL (T4, middle panel) and SIIVFEKL (V4, right panel). Graphs show normalized dose-response values of peptide concentration vs the fraction of maximum numbers of residual DP (CD4 and CD8 bright) thymocytes.

We reasoned that the DO11.10 and HY TCR transgenic models might be inadequate to assess the effect of the R620W mutation on thymic selection if a sufficiently intense stimulation of the TCR is able to overcome the inhibitory effect of the R620W gain-of-function on signaling. We therefore also utilized altered peptide ligands to test how the R620W variant impacts negative selection in a qualitative and quantitative manner. We co-cultured non transgenic, TgLYPW, and TgLYPW^C227S^ OT-1 thymocytes with irradiated target cells and increasing concentrations of either native OT-1 peptide (SIINFEKL, N4) or altered peptide ligands (SIITFEKL, T4 and SIIVFEKL, V4). The latter peptides provide a much lower level of stimulation to OT-1 T cells than SIINFEKL [43]. Exposing double positive thymocytes to a stimulating peptide causes a strong down-regulation of CD4 and CD8 expression [44]. As expected, compared to the wild-type ligand N4, higher concentrations of T4 and V4 were needed to induce co-receptor down-regulation but interestingly, identical dose-response curves were obtained when non transgenic, TgLYPW or TgLYPW^C227S^ OT-1 thymocytes were exposed to the different peptides. We concluded that expression of the two different forms of LYP does not change antigen-sensitivity of OT-1 thymocytes (**Fig. 5**).

**Figure 6:**
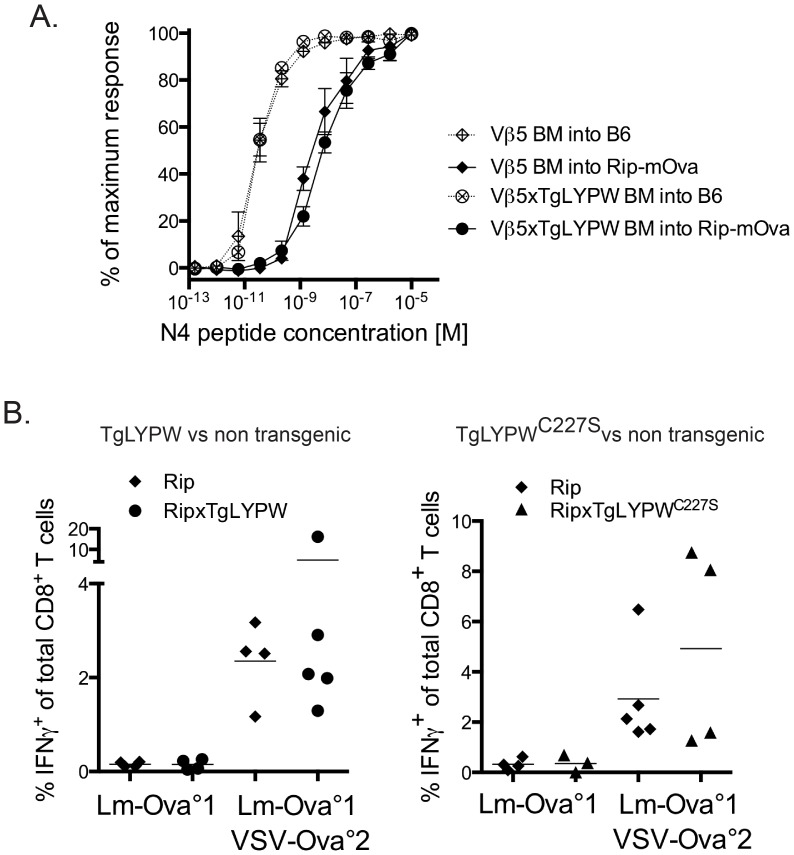
Polyclonal thymocytes undergo similar levels of negative selection in the presence of absence of transgenic LYP-W620. A, Lethally irradiated Rip-mOva mice (continuous graphs and black symbols) or C57BL/6 mice (dotted graphs and crossed symbols) were reconstituted with bone marrow harvested from Vβ5xLYPW (circles) or Vβ5 control (diamonds) mice. 10 weeks after the reconstitution the mice were infected with a strain of *Listeria monocytogenes* expressing Ovalbumin (Lm-Ova). Splenocytes were harvested at 8 days after the infection and briefly *in vitro* re-stimulated with titrated doses of SIINFEKL peptide. Afterwards, the cells were intracellularly stained for IFNγ. Peptide-dose response curves showing the frequency of IFNγ producing CD8^+^ T cells as fraction of maximum response are presented. B, TgLYPW, TgLYP^C227S^ and control non-Tg mice contain similar numbers of low avidity auto-reactive T cells. RipxTgLYPW (left panel, black circles), RipxTgLYPW^C227S^ (right panel, black triangles) and control Rip mice (left and right panels, black diamonds) were infected with Lm-Ova and 4 weeks later challenged by a strain of *Vesicular stomatitis virus* expressing Ova (VSV-Ova). On day 6 after the primary (left side of each panel) or the secondary infection (right side of each panel) blood was drawn from the mice and PBMC were briefly re-stimulated with SIINFEKL peptide. The number of IFNγ producing CD8^+^ T cells was determined by intracellular cytokine staining. Panels show the frequency of Ova-specific T cells.

Next, we assessed the effect of the LYP-W620 transgene on *in vivo* negative selection of polyclonal antigen-specific T cells using a previously established Rip-mOva and Vβ5 transgenic mouse model system [45]. Vβ5 mice express the same TCRβ chain as OT-1 T cells while they re-arrange endogenous TCRα chains [46]. Fixing TCRβ strongly elevates the frequency of K^b^/Ova-specific T cells; however, these T cells cover a wide range from low to high avidity recognition for the Ova-antigen. Crossing Vβ5 to Rip-mOva mice causes the thymic deletion of high avidity Ova-reactive T cells while low avidity T cells are spared [45]. Similar results can be obtained when Vβ5 bone marrow is transferred into Rip-mOva hosts (DZ, unpublished observations). We asked whether TgLYPW could modulate negative selection in Rip-mOva mice and if this manipulation might lead to an escape of higher affinity Vβ5 T cells. Irradiated Rip-mOVA mice were reconstituted with bone marrow from either non-Tg or TgLYPW Vβ5 mice. After infecting these bone marrow chimeric mice with *Listeria monocytogenes*-Ova, we found that T cells with comparable functional avidity for K^b^/Ova could be found in non-Tg or TgLYPW mice (**Fig. 6A**). Since higher avidity T cells normally overgrow lower avidity T cell clones [43], we concluded from our data that there is no change in the negative selection threshold caused by the presence of human LYP-W620. We also assessed whether LYP-W620 expression would result in enhanced expansion of peripheral autoreactive T cell numbers that are normally subject to negative selection in Rip-mOva mice. As previously described, we applied two consecutive infections to expand low avidity clones in Rip-mOva mice [47]. We observed that both normal and LYPW Rip-mOva mice contained similar numbers of low avidity K^b^/Ova-reactive T cells (**Fig. 6B**). Together, the data suggest that active LYP expression does not affect negative selection of thymocytes or the frequency of autoreactive thymic emigrants.

### No Alterations of CD4^+^ Thymocyte Repertoire and Severity of Arthritis in SkgxTgLYPW Mice

**Figure 7:**
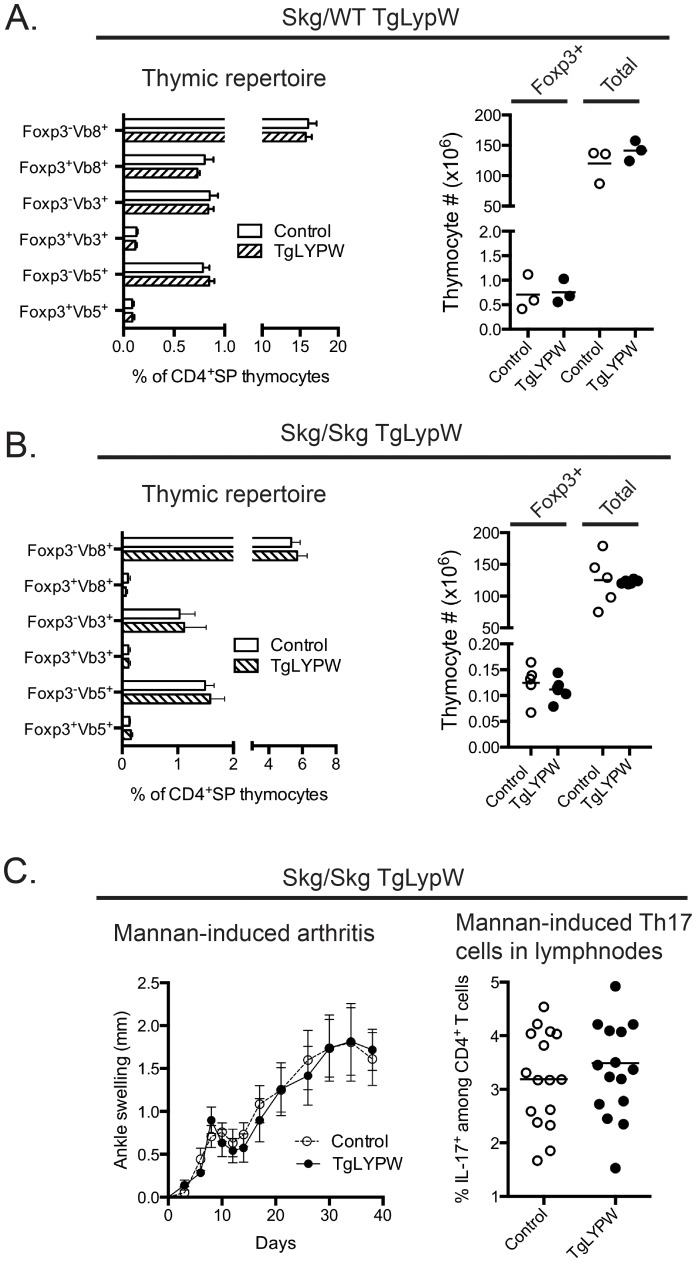
LYPW overexpression does not alter thymic repertoire and autoimmune phenotype of Skg mice. A–B, Overexpression of LYPW does not alter Vβ repertoire or numbers of CD4^+^Foxp3^+^ thymocytes in Skg/WT or Skg/Skg mice. Left panel shows average and SE % Vβ positive CD4^+^Foxp3^+^ and CD4^+^Foxp3^−^ thymocytes from Skg/WT (A) or Skg/Skg (B) TgLYPW (striped bars, n = 3 for Skg/WT and n = 6 for Skg/Skg) and control non-Tg littermates (white bars, n = 3 for Skg/WT and n = 5 for Skg/Skg) as assessed by flow cytometry analysis after staining with anti-Vβ3, -Vβ5 and -Vβ8 antibodies. Right panel shows mean and range of CD4^+^Foxp3^+^ (first and second bar) and total (third and fourth bars) thymocytes from the same Skg/WT (A) or Skg/Skg (B) TgLYPW mice (striped bars) or non-Tg littermates (white bars). C, Overexpression of LYPW does not alter the course of mannan-induced arthritis and the frequency of Th17 cells in peripheral lymph node (LN) of arthritic Skg mice. Left panel shows arthritis score (measured as ankle swelling in mm) of Skg/Skg TgLYPW mice (black circles, n = 6) and littermates non-Tg Skg/Skg mice (white circles, n = 5) followed-up for 40 days after a single i.p. injection of 20 mg mannan dissolved in 200 µl PBS. One month following mannan-injection, LN cells from Skg/Skg TgLYPW mice (black circles, n = 15) or non-Tg Skg/Skg littermates (white circles, n = 16) were stimulated with 20 ng/ml PMA and 2 mM ionomycin for 5 hours. Right panel shows % Th17^+^ cells of the CD4^+^ T cell population as assessed by flow cytometry analysis after intracellular staining with an anti-IL17 antibody.

In order to assess the effect of LYPW overexpression on the thymic repertoire of CD4^+^ T cells, we crossed TgLYPW mice with Skg mice. Skg mice carry a mutation in *Zap70* (W163C) that attenuates T cell receptor signaling [35]. However, to some extent, the reduced function *Zap70* mutant can be compensated such that CD4^+^ and CD8^+^ T cells can be found in the periphery of Skg mice. Interestingly, a chronic form of arthritis develops spontaneously in these mice. Moreover, disease can be induced by injecting fungal-derived polysaccharides into animals that are homozygous for the Skg mutation and express at least one copy of the H2^d^ haplotype ( [35], [39], [40] and our unpublished data). It has also been shown that Skg/Skg and heterozygote Skg/WT mice display alterations in the Vβ repertoire usage by CD4^+^Foxp3^−^ thymocytes and even more markedly in CD4^+^Foxp3^+^ thymocytes, which are supposedly skewed toward a more self-reactive repertoire. These alterations include decreased deletion of Vβ3^+^ and Vβ5^+^ T cells and decreased positive selection of Vβ8^+^ T cells [37]. Qualitatively similar but quantitatively more marked alterations of the CD4^+^ Vβ repertoire, correlating with a more severe decrease in thymocyte TCR signaling and thymocyte selection, were found in homozygous Skg/Skg animals. Thus, we reasoned that Skg/WT and Skg/Skg animals would be a sensitive model to detect subtle alterations in CD4^+^ T cell selection caused by decreased thymocyte TCR signaling [36], [37]. **Fig. 7A and 7B** show that no alterations of the frequencies of Vβ3^+^, Vβ5^+^, or Vβ8^+^ thymocytes were found among CD4^+^Foxp3^−^ or CD4^+^Foxp3^+^ thymocytes in Skg/WT or Skg/Skg TgLYPW mice versus non transgenic littermates, suggesting that the decrease in TCR signaling caused by the active phosphatase transgene is insufficient to cause significant alterations in CD4^+^ T cell selection. Accordingly, the LYPW transgene did not cause increased severity of mannan-induced arthritis in Skg/Skg mice (**Fig. 7C**), and equal numbers of lymph node Th17 cells were found in Skg/Skg TgLYPW and littermate Skg/Skg mice one month after induction of arthritis by intraperitoneal injection of mannan (**Fig. 7C**).

## Discussion

With respect to genetic effect potency, the LYP-R620W polymorphism currently ranks in second and third position, respectively, as a risk factor for RA and for type 1 diabetes [2], [48]. Although it is evident that the R620W variation is one of the major non-HLA genetic risk factors for autoimmunity, its mechanism of action at the molecular level and its role in the immunopathogenesis of disease remain unclear.

Our experiments in primary T cells from type 1 diabetes subjects and in Jurkat and primary human T cells overexpressing LYPW lead us to propose a model that LYP-W620 acts as a gain-of-function variant in phosphatase activity and in negative regulation of TCR signaling [12]. Accordingly, an initial hypothesis as to how the R620W variation might promote autoimmunity was that variant LYP augments inhibition of thymocyte TCR signaling and allows the escape of higher numbers of auto-reactive T cells or of T cells exhibiting a higher functional avidity (higher strength of self-pMHC and TCR interaction) [12], [49]. Since formulation of the hypothesis, additional data variably supporting a “gain-of-function”[13], [15]–[18], “loss-of-function” [20], [21], [50] or “altered-function” [23], [24] phenotype of LYP-W620 in TCR signaling have been published.

*Ptpn22*-deficient mice exhibit abnormal accumulation of effector-like and memory T cells and heightened lymphoproliferation capacity without clear-cut breaches in peripheral tolerance [9], but it has remained unclear whether these phenotypes solely relied on a lack of peripheral or thymic Pep expression. Two independent studies have found evidence of increased qualitative positive thymocyte selection in *Ptpn22* KO mice transgenic for the D011.10 [9] and OT-II TCRs [10]. Another group recently overexpressed WT Pep under control of the T lineage specific distal *Lck* promoter [27], and found no alterations in thymocyte subpopulation numbers in transgenic vs non transgenic mice. However, based on the published *Ptpn22* KO, knock-in, or overexpression studies, no prediction could be made how a presumed gain of function R620W variant of human LYP might impact negative selection of auto-reactive T cells. Our study was designed to test the hypothesis that the gain-of-function inhibition of TCR signaling by LYP-W620 in thymocytes is sufficient to alter thymic output in a manner that could increase predisposition to autoimmunity. We studied the human phosphatase variant rather than its mouse homolog W619 because it is currently unclear whether regulation by Csk is conserved between the human and mouse phosphatase, or whether the gain-of-function nature of the R620W variant is fully replicated by the R619W mutation of mouse Pep. To detect possible enzymatic activity-independent effects of the phosphatase, we also generated animals transgenic for the inactive C227S mutants of LYP-W620. In the presence of LYP-W620, we observed increased phosphatase activity of LYP-W620 in thymocytes, associated with decreased TCR signaling in total thymocytes and DP thymocytes. No anomalies in thymic TCR signaling were detected in animals carrying the inactive form of LYP-W620, despite supraphysiological expression of LYP directed by the transgene in these animals. Results with LYP-W620 C227S variant-bearing mice thus suggest that effects of phosphatase overexpression on thymocyte TCR signaling critically depend upon enzymatic function of LYP.

Overexpression of active LYP-W620 did not lead to impaired positive or negative selection of HY-TCR transgenic thymocytes in male mice or in OT-1 TCR-transgenic thymocytes crossed to Rip-mOva mice. Indeed, the finding of modestly-reduced autoreactive thymocyte numbers in BACLYP (human *PTPN22* promoter-driven) transgenic H-Y males suggests that LYP-W620 may actually promote negative selection of CD8^+^ T cells. Such a phenotype is in line with the report by Dai *et al.*, in which Pep-W619-knockin autoreactive thymocytes exhibit augmented clonal deletion [23]. We speculate that such subtle positive effects of LYP-W620 on thymocyte selection are obliterated in our TgLYP models by the combination of supraphysiological expression levels and the use of an exogenous promoter.

Next, we considered the hypothesis that overexpression of the LYP-W620 variant would only impact negative selection at an intermediate TCR affinity range and thus might particularly impact those clones bearing TCR with affinity for self-antigen close to the threshold of negative selection. We addressed this hypothesis by comparing the affinity of the bulk population of K^b^/Ova-reactive T cells that survive negative selection in Rip-mOva mice. Normally, in such mice only T cells with low affinity for K^b^/Ova can be detected in the periphery [45]. We reasoned that if LYP-W620 selectively reduces negative selection of T cells bearing antigen receptors with affinity at or just above the threshold of negative selection, then expression of TgLYPW would confer increased in the overall functional avidity of K^b^/Ova-reactive T cells in Rip-mOva or Rip-mOvaxVβ5 mice. Our negative data clearly argue against this hypothesis.

We also considered whether LYP-W620 might selectively impact CD4^+^ T cell selection. The pathogenesis of disease in Skg mice depends on reduced TCR signaling in thymocytes, which results in reduced positive and negative selection of CD4^+^ thymocytes, but the repertoire of Skg thymic emigrants contains T cells with significant auto-reactive potential [36], [37]. When we overexpressed LYP-W620 in the thymus of Skg mice, we did not observe alterations of CD4^+^ thymocyte repertoire or a gain in disease severity. Higher affinity interactions with thymocyte TCR by autoantigens are thought to be required for the differentiation of T_reg_ in the thymus, thus suggesting that alterations of TCR signaling in the thymus might shape the amount and repertoire of T_reg_ [51]. However, we did not see changes in the output of regulatory T cells from the TgLYPW Skg thymus, further arguing against the notion that LYP-W620 selectively affects high-affinity TCR interactions important for development.

Importantly, in the TgLYPW model, expression of the transgene is controlled by the *Lck* proximal promoter and is only expressed in the thymus but not in the periphery. Therefore, we can exclude that we created a balanced situation such that we might have augmented the thymic threshold but, at the same time, caused similar increases in the peripheral T cell activation threshold. Altogether, our observations imply that LYP-W620 does not have a major negative impact on selection of T cells in general or on the threshold of negative selection in mice. This is well in line with observations that complete absence of Pep had no impact on negative selection [9].

Given the lack of impact on thymic selection, we suggest that LYP-W620 might primarily act on peripheral T cells. A selective effect of LYP-W620 on signaling in peripheral T cells would be consistent with the observed increase of effector/memory T cells in Pep KO mice. However, a gain of function mutant that increases the activation threshold of effector T cells is difficult to reconcile with auto-immunity. We therefore consider that a peripheral model of promoting auto-immunity through the LYP-R620W mutation could involve an impaired T_reg_ function. However, others have not documented diminished T_reg_ function in 619W knock-in mice [23].

Our findings lead us to the conclusion that the mechanism by which LYP-W620 impinges on the immunopathogenesis of T cell-modulated human disease is still uncertain and does not involve major effects on thymocyte-intrinsic processes that establish central tolerance. LYP-W620 might involve yet unknown effects on T cells or it might cause a rather subtle impact that we failed to detect in our experimental systems. One study reported that subjects carrying LYP-W620 have increased numbers of effector T cells [15]. A recent report also suggests that the R620W variation might have unknown “change-in-function” effects [24] on T cells.

The LYP-R620W variation might impact the function of other cell types including B cells. It has been suggested that an increased burden of autoreactive B cells –perhaps secondary to weakened B cell negative selection– underlies the predisposition of LYP-W620-carrying subjects to autoimmune diseases [52]. Zhang *et al*. found increased activation of dendritic cells in mice carrying a Pep-R619W knock-in mutation, suggesting that hyperactive myeloid cells also might also contribute to the mechanism of action of the autoimmune predisposing variant [21]. Notably, the complexity and subtlety of LYP-W620 function in myeloid cells likely rivals that of its action in lymphocytes. Our recent work revealed selective loss of capacity to produce type 1 interferon among dendritic cells derived from BACLYPW transgenic mice [31]. Such a myeloid cell defect could contribute to cell-extrinsic aberrations in effector T cells responding to infection or inflammation, yet be consistent with absence of a T cell developmental phenotype for LYP-W620.

Our results are supported by experiments in two different mouse transgenic systems and multiple monoclonal and polyclonal TCR transgenic models. However, there are few limitations, including 1) the use of an overexpression system, which is amenable to positional effects, 3) although knock-down and deletion experiments suggest that the function of the phosphatase in TCR signaling is conserved in human and mouse cells, the expression of a human phosphatase in a mouse context can be viewed as another limitation of our model and 4) the use of hybrid mouse strains in some experiments might have decreased the sensitivity to small effects. Despite the above-mentioned limitations, our data strongly suggest that the increased activity of LYP, which –according to several groups– is conferred by the R620W variation, is insufficient to cause anomalies in thymic selection that could underlie the increased risk of autoimmunity conferred by carriage of the variant. TgLYPW mice transgenic for the active phosphatase variant carried an average calculated phosphatase activity between those reported for heterozygous or homozygous human carriers of the LYP-W620 variant. Since the overall overexpression of the phosphatase was low, the most likely explanation for the lack of a phenotype is that the decrease in thymocyte TCR signaling caused by the overexpression of the phosphatase was too small to significantly affect thymic selection. An additional or alternative explanation to explain the lack of phenotype in SkgxTgLYPW mice is that after the threshold of signaling inhibition necessary to trigger thymic signaling anomalies has been trespassed, quantitative reductions in signaling are required in order to see further decreases of selection and an increase in severity of arthritis. Since no studies to date have uncovered profound effects of LYP-W620 on thymic selection, an alternative explanation is that TCR signaling in thymocytes might be controlled by multiple redundant phosphatases. LYP-dependent effects might not be detectable in the presence of a dominant regulator such as CD45, which plays a major role in both positive and negative regulation of TCR signaling in double positive and single positive thymocytes [53].
